# Aseptic meningitis, hepatitis and cholestasis induced by trimethoprim/sulfamethoxazole: a case report

**DOI:** 10.1186/s12887-021-02820-y

**Published:** 2021-08-16

**Authors:** J. A. A. van Asperdt, R. A. De Moor

**Affiliations:** Department of Paediatrics, Elisabeth-Tweesteden Hospital, Hilvarenbeekseweek 60, 5022 GC Tilburg, The Netherlands

**Keywords:** Case report, Aseptic meningitis, Hepatitis, Drug-induced, Trimethoprim-sulfamethoxazole

## Abstract

**Background:**

Drug-induced aseptic meningitis is a rare, but challenging diagnosis, most commonly reported with nonsteoroidal anti-inflammatory drugs (NSAIDs) and antibiotics. Trimethoprim/sulfamethoxazole (TMP/SMX) is a sulfonamide that is widely used in clinical practice for the treatment and prophylaxis of various infections. The most common side effects associated with TMP/SMX are generally mild and self-limited, but serious side effects have been reported, including liver injury and aseptic meningitis.

**Case presentation:**

We report a 2,5 year old Dutch girl with both drug-induced aseptic meningitis and drug-induced liver injury while using TMP/SMX prophylaxis. Ursodeoxycholic acid was started because of cholestatic injury. After cessation of TMP/SMX, full convalescence was reached within weeks.

**Conclusions:**

This is the first report of a young patient with both aseptic meningitis and drug-induced liver injury caused by TMP/SMX. Drug-induced aseptic meningitis and cholestatic hepatitis constitute a considerable diagnostic challenge to clinicians. In addition to a thorough evaluation for infectious causes, clinicians should be aware of drug-induced aseptic meningitis and cholestatic hepatitis.

**Supplementary Information:**

The online version contains supplementary material available at 10.1186/s12887-021-02820-y.

## Background

Drug-induced aseptic meningitis is a known adverse reaction to some drugs, including antibiotics. A frequently implicated antibiotic is trimethoprim-sulfamethoxazole (TMP/SMX). Several cases of TMP/SMX aseptic meningitis have been reported in literature and (as individual case safety reports) to pharmacovigilance databases for drug monitoring. In pediatric patients, only a few cases have been reported, with the youngest being 3 years old [1–5]. Another known adverse effect of TMP/SMX, even more rare in children, is drug-induced liver injury. In recent literature only one pediatric case has been published with both drug-induced aseptic meningitis and hepatitis, reporting a 16-year old girl using TMP/SMX together with valproic acid [4]. To our knowledge, our case is the first report of a young patient with TMP/SMX-induced aseptic meningitis and hepatitis without other drugs involved. Drug-induced aseptic meningitis or cholestatic hepatitis are a considerable diagnostic challenge to clinicians, as shown by the presented case.

## Case presentation

A 2.5 year old girl was admitted to the hospital on suspicion of a meningitis. Because of recurrent otitis prophylactic TMP/SMX was initiated 2 weeks prior to admission. She presented with fever, intermittent drowsiness, crying and positive Kernig’s sign. Blood tests showed normal white blood cell count and C-reactive protein. The cerebrospinal fluid (CSF) analysis showed pleocytosis (41 x 10^6 WBC/L, 100% mononuclear cells), normal glucose and protein levels. CSF gram stain showed no bacteria. With a suspicion of viral meningitis aciclovir was started intravenously. TMP/SMX prophylaxis was continued and switched into intravenous administration because of poor intake. In the following days symptoms of meningitis did not improve. CSF culture and PCR on mumps virus, enterovirus, parechovirus, herpes simplex and varicella zoster virus were negative. Blood culture was also negative. Because of the common association of aseptic meningitis and some autoimmune diseases, antinuclear antibodies (ANA) were measured, which were negative [6].

On the fourth day of admission she developed jaundice. The laboratory tests showed a conjugated hyperbilirubinaemia (total 76 μmol/L , conjugated bilirubin 74 μmol/L) with increased transaminase levels (AST 332 U/L, ALT 358 U/L). An ultrasound of the liver and gallbladder was normal. Serology on EBV, CMV, Hepatitis A, B, C and E, Bartonella Henselae and Borrelia Burgdorferi were negative. Immune globulin serum levels (IgA, IgG and IgM) were normal. Markers for auto-immune hepatitis (ASMA, AMA, ANCA, anti-LKM-1, Anti-DNA antibodies) for hepatitis were negative.

**Fig. 1:**
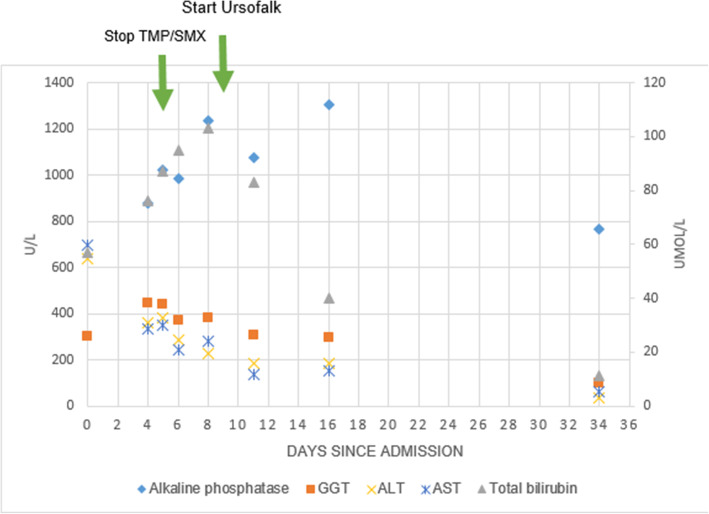
Follow-up laboratory testing (Bilirubin on right axis. APT, GGT, ALT, AST on left axis)

After exclusion of infectious or auto-immune causes drug-induced aseptic meningitis with cholestatic hepatitis was considered and the TMP/SMX and Aciclovir treatment were discontinued. Because of increasing cholestasis we initiated urosodeoxycholic acid therapy on the 9^th^ day of admission. The dynamics of parameters for hepatitis are depicted in Fig. 1.

The clinical condition of the child improved. After cessation of the TMP/SMX, symptoms of meningitis quickly resolved. Two weeks after her admission she was discharged from the hospital in good clinical condition. She reached convalescence for both clinical and laboratory parameters in a few weeks.

## Discussion and conclusions

Aseptic meningitis is a rare entity, although the exact incidence is still uncertain. Many cases remain unrecognized, since it constitutes a diagnostic challenge. Nonsteroidal anti-inflammatory drugs (NSAIDs) are the most commonly identified causative agent. TMP/SMX is the most commonly observed antimicrobial drug causing aseptic meningitis [6, 7]. A recent literature review studied 41 cases of aseptic meningitis caused by TMP/SMX, reporting 5 pediatric patients up to 18 years [1]. The youngest patient reported in this review was 6 years old. Most patients developed symptoms within 24 hours after first exposition to the drug, but one patient had been on TMP/SMX for 3 months before developing symptoms. Our patient was taking prophylactic TMP/SMX for 2 weeks before symptoms initiated.

The exact pathogenic mechanism is still unknown. The clinical pattern of injury with TMP-SMX suggests a drug allergy or hypersensitivity mechanism, perhaps through its metabolism to a toxic, reactive or antigenic metabolite. It has been proposed that the drug would form a hapten conjugation with a CSF or meningeal protein, being a type II or type III hypersensitivity reaction. There are cases of TMP/SMX-induced aseptic meningitis in which immune complexes have been found in plasma, but not in CSF [7]. Another mechanism taken into consideration is related to type IV hypersensitivity, using the T-cell by reversibly binding to the human leukocyte antigen (HLA) on its receptor [8].

Drug-induced aseptic meningitis is a diagnosis of exclusion, made after infectious causes have been ruled out. A temporal relationship between the use of the drug and subsequent onset of meningeal symptoms, negative CSF cultures/PCR on viruses, and resolution of symptoms after drug withdrawal are the key to diagnosis. In our case the combination of aseptic meningitis and cholestatic hepatitis without an infectious causative agent raised consideration of a drug-induced condition.

Drug-induced liver injury is a known adverse effect of many drugs. 2-5% of all patients presenting with jaundice are diagnosed with drug-induced liver injury [9]. It is most reported in adults and antibiotics (especially amoxicillin-clavulanate) are the main cause world-wide. In children only valproic acid is well known to be causing liver injury, other agents are rare.

TMP/SMX is known to cause idiosyncratic liver injury, which is not typically dose-related, is unpredictable (happens in only a small proportion of exposed individuals) and has a variable latency to onset [10]. The interval can be ranging between 2-12 weeks from the initial ingestion of the drug [11]. In our case the interval was 2.5 weeks.

Drug-induced liver injury also is a diagnosis of exclusion, made after infectious and auto-immune causes have been excluded. We decided to start ursodeoxycholic acid when laboratory results further deteriorated after cessation of the TMP/SMX. Literature studies state that the efficacy may not be substantiated. It is not well documented, no controlled studies have been published and the studies that have been published show contradicting results [10]. In 2013 a case report affirms a progressive improvement with ursodeoxycholic acid. According to consensus by the Council for International Organizations of Medical Sciences (june 2020) initiation of ursodeoxycholic acid is an option under consideration and requires further study [12, 13]. We decided to continue ursodeoxycholic acid in outpatient setting. Full convalescence was reached in a few weeks. TMP/SMX-induced aseptic meningitis, as well as TMP/SMX-induced hepatitis have a good outcome with slowly normalisation of laboratory results [11, 14].

A drug challenge can confirm the diagnosis of drug-induced aseptic meningitis, but is considered unethical because of reported increasing symptoms after re-exposure [1, 7, 10, 11]. In patients with an unconvincing clinical history experiencing only mild symptoms or in cases with no alternative antibiotic choice and the risk outweighs the benefit, a drug rechallenge could be considered [1].

Clinicians should be aware of the possible severe consequences of (prolonged) TMP/SMX use and have a high index of suspicion of drug-induced aseptic meningitis in cases with symptoms of meningitis and negative CSF culture, especially in the combination with liver injury. The symptoms of drug-induced aseptic meningitis usually resolve quickly after cessation of causative agent. Further research is needed to identify the exact mechanism of the reaction.

## Supplementary Information

**Additional file 1.** CARE checklist.

## Data Availability

Data sharing is not applicable to this article as no datasets were generated or analysed during the current study.

## Abbreviations

TMP/SMX: Trimethoprim-sulfamethoxazole
ANA: Antinuclear antibodies
ANCA: Anti-neutrophilic cytoplasmic autoantibody
ASMA: Anti-smooth muscle antibody
AMA: Anti-mitochondrial antibody
Anti-LMK-1: Anti-liver kidney microsome type 1
HLA: Human leukocyte antigen
CSF: Cerebrospinal fluid
NSAIDs: Nonsteroidal anti-inflammatory drugs
